# Biomedical Science Ph.D. Career Interest Patterns by Race/Ethnicity and Gender

**DOI:** 10.1371/journal.pone.0114736

**Published:** 2014-12-10

**Authors:** Kenneth D. Gibbs, John McGready, Jessica C. Bennett, Kimberly Griffin

**Affiliations:** 1 Cancer Prevention Fellowship Program, Division of Cancer Prevention, National Cancer Institute, Bethesda, Maryland, United States of America; 2 Science of Research and Technology Branch, Behavioral Research Program, Division of Cancer Control and Population Sciences, National Cancer Institute, Bethesda, Maryland, United States of America; 3 Department of Biostatistics, Johns Hopkins Bloomberg School of Public Health, Baltimore, Maryland, United States of America; 4 Department of Counseling, Higher Education, and Special Education, University of Maryland, College Park, Maryland, United States of America; World Health Organization, Switzerland

## Abstract

Increasing biomedical workforce diversity remains a persistent challenge. Recent reports have shown that biomedical sciences (BMS) graduate students become less interested in faculty careers as training progresses; however, it is unclear whether or how the career preferences of women and underrepresented minority (URM) scientists change in manners distinct from their better-represented peers. We report results from a survey of 1500 recent American BMS Ph.D. graduates (including 276 URMs) that examined career preferences over the course of their graduate training experiences. On average, scientists from all social backgrounds showed significantly decreased interest in faculty careers at research universities, and significantly increased interest in non-research careers at Ph.D. completion relative to entry. However, group differences emerged in overall levels of interest (at Ph.D. entry and completion), and the magnitude of change in interest in these careers. Multiple logistic regression showed that when controlling for career pathway interest at Ph.D. entry, first-author publication rate, faculty support, research self-efficacy, and graduate training experiences, differences in career pathway interest between social identity groups persisted. All groups were less likely than men from well-represented (WR) racial/ethnic backgrounds to report high interest in faculty careers at research-intensive universities (URM men: OR 0.60, 95% CI: 0.36–0.98, p = 0.04; WR women: OR: 0.64, 95% CI: 0.47–0.89, p = 0.008; URM women: OR: 0.46, 95% CI: 0.30–0.71, p<0.001), and URM women were more likely than all other groups to report high interest in non-research careers (OR: 1.93, 95% CI: 1.28–2.90, p = 0.002). The persistence of disparities in the career interests of Ph.D. recipients suggests that a supply-side (or “pipeline”) framing of biomedical workforce diversity challenges may limit the effectiveness of efforts to attract and retain the best and most diverse workforce. We propose incorporation of an ecological perspective of career development when considering strategies to enhance the biomedical workforce and professoriate through diversity.

## Introduction

Increasing the participation of women and scientists from underrepresented minority (URM) backgrounds in the science professoriate remains “perhaps the least successful of the diversity initiatives” [1]. In the biomedical sciences (BMS), women earn more than half of Ph.Ds. but represent 33% of newly hired tenure/tenure-track (TTT) professors. Scientists from URM backgrounds earn 10% of life science Ph.Ds. but represent 2% of medical school basic science TTT faculty—a number unchanged since 1980 [2]–[4]. The benefits of diversity, including enhanced creativity in problem solving [5]–[7] and improved learning outcomes for students from traditionally underrepresented backgrounds [8]–[10] (the latter of which is thought to be particularly important to maintaining long-term US competitiveness in an increasingly diverse society [11]), have lead policy makers to focus anew on increasing diversity in the BMS workforce and professoriate [12], [13].

In the BMS, initiatives to boost faculty and workforce diversity are taking place against a backdrop of systemic disequilibrium [14]. In the decade following the doubling of the NIH budget, available funding for research has declined by up to 25% in constant dollars and success rates for research project grants have also decreased; yet, the system continues to produce greater numbers of Ph.D. scientists than there are permanent research positions in academia, government and the private sector [15]. This has been particularly evident in academic science, where in the early 1970s, over 50% of life science Ph.D. graduates held tenure/tenure-track positions within 5 years of graduation, while today, that number has declined to 10.6% [16], [17].

In line with these structural changes, recent surveys of BMS Ph.D. students have shown that as graduate training progresses, smaller percentages express interest in faculty careers at research-intensive universities, and greater percentages express interest in careers outside of academia and in non-research based careers [18], [19]. While providing valuable insights, these reports have largely left unaddressed the extent to which these career interest patterns vary based on social identity—specifically, race/ethnicity, gender, and their intersection. The professional interests of early-career Ph.D. scientists of all backgrounds are key to the future composition of the workforce as interest represents a necessary (but not sufficient) antecedent to the pursuit and attainment of a career path [20].

While declining interest in academia may be a general trend, it remains unclear whether part of the reason women and URMs are underrepresented in academia results from a differential shift in their desires to pursue faculty work relative to their better-represented peers. This study builds on our previous work examining the mechanisms underlying the career interest formation of recent BMS Ph.D. recipients [21], and the above mentioned surveys of the career preferences of BMS graduate students, by addressing three questions:

1. Are there distinct career interest patterns based on social identity (race/ethnicity, gender and their intersection) in recent BMS Ph.D. graduates?
2. To what extent do personal dispositions (e.g. initial career interest and research self-efficacy), objective measures (e.g. rate of first-author publications, institution type), and graduate training experiences (e.g. sense of belonging, advisor interactions, career development) predict interest in academic careers at Ph.D. completion?
3. Do any differences in career interests across social identity remain after accounting for personal dispositions, research self-efficacy, objective performance measures, and graduate training experiences?

## Materials and Methods

### Data Collection and Procedures

The study was done in compliance with and approved by the University of Maryland Institutional Review Board (IRB # 373799-5). All participants consented to participation in the study. A purposeful sampling strategy [22] was developed to recruit a diverse set of participants with respect to social identity (i.e., race/ethnicity and gender) through: listservs of Ph.D.-level, science-policy professionals and academic and government postdoctoral scientists; direct contact at national scientific conferences; administrators at US research universities and in companies that train postdoctoral scientists; and through the “STEM PhD Careers” LinkedIn and Twitter accounts managed by the investigators. Participants were also asked to forward the study notice to recruit other eligible peers (i.e., snowball sampling [23]).

All participants completed a short survey on their graduate and postdoctoral training experiences, career development, and professional interests (S1 Figure). The instrument was developed utilizing themes emerging from our previous work [21], as well as the instruments used by Fuhrmann et al., and Sauermann and Roach [18], [19]. The online survey link was available from October 2012 – January 2013, and all responses were collected using the software suite Qualtrics (www.qualtrics.com). The sampling strategy yielded 1890 complete, unique responses. From the 1890 remaining responses, those who indicated completion of a Ph.D. in the biomedical and behavioral sciences (as defined by the NIH Biomedical Workforce Report [24]) between 2007-2012 were included for analysis in this study (n = 1500).

### Career Interest Measures and Statistical Analysis

Respondents were asked to rate their interest in pursuing each of the following career pathways at three time points: (i) the beginning of their Ph.D. training, (ii) the completion of their Ph.D. training, and (iii) currently. These pathways were:

- Faculty at a research-intensive university
- Faculty at a teaching-intensive university
- Research career, non-academic (e.g. industry, pharmaceutical, biotech, government, start-up, etc.)
- Non-research career (e.g. consulting, policy, science writing, patent law, business, etc.)

Interest was measured on a six-point scale where 0 represented not knowledgeable, 1 no interest, 2 low interest, 3 moderate interest, 4 interest, and 5 strong interest. For analytic purposes, respondents answering not knowledgeable were recoded as having no interest. Sensitivity analysis was performed and inferences regarding change in career interest, or differences in career interest profile between social identity groups, did not change based on this reclassification.

### Social Identity

Social identity was stratified based on the intersections of race/ethnicity and gender. Definitions of race-ethnicity were consistent with those utilized for federal designation [25]. Participants were classified as belonging to a “well-represented” (WR) racial-ethnic group if they identified their racial/ethnic identity as “White,” “Asian/Asian American,” or both “White” and “Asian/Asian American” based on the proportion of scientists and engineers (S&E) from these backgrounds working in S&E occupations [4]. Participants were classified as belonging to an “underrepresented minority” (URM) group if they selected any of the following racial/ethnic categories: “American Indian/Alaska Native,” “Black/African-American,” “Hispanic/Latino,” or “Native Hawaiian/Pacific Islander,” consistent with NIH definition [26]. Males from well-represented backgrounds are referred to as WRM (25% of sample); males from underrepresented minority backgrounds are referred to as URMM (5.8% of sample); females from well-represented backgrounds are referred to as WRF (53% of sample); and females from URM backgrounds are referred to as URMF (12.6% of sample).

### Statistical Analysis

Paired t-tests were used to assess intra-individual and intra-group changes in level of career pathways interest across time points [27]. In comparing level of interest between social identity groups at any time point, Bonferroni-corrected ANOVA was utilized. All statistical analysis was conducted using Stata 13.0, and figures were made using GraphPad Prism and Adobe Illustrator.

Multiple logistic regression was utilized to determine the relationship between covariates previously linked to interest in various career pathways at Ph.D. completion. Career interest measures (at Ph.D. entry and Ph.D. completion) were dichotomized into high interest (i.e. 4-5), and those without high interest (1-3), and standard errors were adjusted to account for the potential clustering of responses by academic institution. The variable of interest, social identity, was coded using 3 indicator variables (URMM, WRF, and URMF) with WRM as the reference group. Additional predictors included in the regression analysis were: personal dispositions, objective & performance measures, and graduate training experiences.

Personal dispositions included dichotomized interest in the career pathway at Ph.D. entry, intentions to pursue a faculty career at Ph.D. entry, and confidence in one's ability as an independent researcher (measured on a 5-point agreement scale where 1 was “strongly disagree” and 5 was “strongly agree”). Objective performance measures included first-author publication rate (first-authored publications/total years in graduate training, postdoctoral training and (when applicable) faculty position), h-index [28], time-to-Ph.D. completion (self-reported), and completion of a Ph.D. at one of the top 50 research universities. Finally, graduate training and career development measures were measured on a 5-point agreement scale and included the extent to which participants felt that they belonged intellectually and socially to their graduate research group and graduate department, had graduate advisor investment in their career, had structured career development offered by their graduate department, and had support for multiple career paths (academic and non-academic) from their graduate advisor and graduate department.

### Limitations

There are a number of limitations to this work. This is not a random sample, and may limit generalizability of the findings. Moreover, we rely on self-reported measures of career interest and training experiences, and respondents may attempt to provide answers that are socially acceptable. We attempted to minimize this by indicating that their identities and responses would be kept strictly confidential, as was done in comparable work [18], [19]. Additionally, we asked the respondents to retrospectively assess their career interests, and training experiences, introducing the potential for recall bias. That said, it is important to account for individuals' understandings of their experiences as one's perceptions of their experiences are linked to measurable educational outcomes such satisfaction, persistence, and academic achievement [29]–[31]. Although our sampling strategy does not permit the calculation of a formal response rate, the sample included here represents approximately 4.7% of eligible respondents (i.e. American biological sciences Ph.Ds. awarded between 2007–2012), and 10.3% of eligible Ph.D. scientists from URM backgrounds in this category [4]. This represents, to our knowledge, the largest sample of scientists from URM backgrounds in the past decade [32], and an important contribution to the discussions of workforce development and diversity.

## Results

### Ph.D. Scientists Show Decreased Interest in Academia and Increased Interest in Non-Research Careers

The 1500 survey respondents were US citizens and permanent residents who completed their Ph.D. in the biomedical sciences between 2007–2012. These scientists trained at 184 different US institutions, and 64.5% of the respondents completed their Ph.D. at one of the top 50 research universities (with respect to science & engineering research and development expenditures [17]) (S1 Table). Two-thirds of respondents worked as postdoctoral scientists (66.8%), with others working in careers outside of research (e.g. science policy, science communication, law, or business; 9.2%), as research scientists/engineers in industry or government (5.7%), tenure-track professors (4.1%), or other positions in academia (4.1%; S2 Table). The sample also included Ph.D. scientists from a wide variety of biomedical disciplines. The five largest fields were: biochemistry/cellular and molecular biology (30%), neuroscience (13.2%), microbiology & immunology (12.7%), pharmacology/toxicology (7.4%), and psychology (7.4%) (S3 Table). Thus, this sample represents a diverse group to evaluate career decisions and training experiences. Additional descriptive data on the sample can be found in S1-S3 Tables, and the survey instrument can be found in S1 Figure.

**Figure 1:**
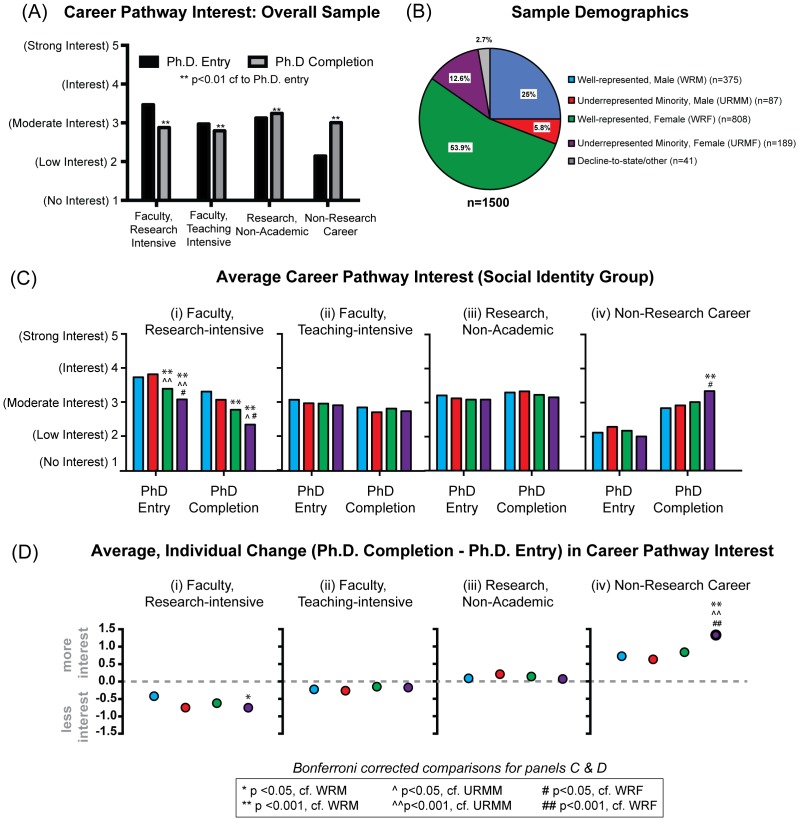
Distinct career interest profiles among Ph.D. biomedical scientists by social identity. (A) Bar graph showing mean response for sample of 1500 American biomedical scientists who received Ph.Ds. between 2007–2012 when asked to rate their level of interest in each of the following career paths at Ph.D. entry (black), Ph.D. completion (grey), on a 5-point scale (where 1 represents “no interest” and 5 represents “strong interest”): faculty at a research-intensive university; faculty at a teaching intensive university; a research career outside of academia (e.g. industry, pharmaceutical, biotech, government, start-up, etc.); and a non-research career (consulting, policy, science writing, patent law, business, etc.). (B) Pie chart showing the social identities of the respondents. Males from well-represented racial/ethnic backgrounds (WRM) are shown in blue and represent 25% of the sample; males from underrepresented minority backgrounds (URMM) are shown in red and represent 5.8% of the sample; females from well well-represented racial backgrounds (WRF) are shown in green and represent 53.9% of the sample; females from URM backgrounds (URMF) are shown in purple and represent 12.6% of the sample; and respondents declining to state racial/ethnic background or with an alternative gender identification are shown in grey and represent 2.7% of the sample. (C) Bar chart showing mean interest in the four career paths at Ph.D. entry, Ph.D. completion across social identity. Group means were compared at each time point and statistical significance was determined using Bonferroni corrected ANOVA. (D) Plot showing the average, individual level paired-difference between career pathway interest at Ph.D. completion versus Ph.D. entry across social identity groups. Statistical significance was determined using Bonferroni corrected ANOVA.

Scientists described their level of interest at Ph.D. entry and Ph.D. completion (on a 5-point scale where 1 represented “no interest”, and 5 represented “strong interest”) in four career pathways: (i) faculty at a research-intensive university, (ii) faculty at a teaching-intensive university, (iii) a research career outside of academia, (e.g. industry, pharmaceutical, biotech, government, or a start-up), or (iv) a non-research career, (e.g. consulting, policy, science writing, patent law, or business). Responses largely mirrored those of graduate students in previous research [18], [19]. There were significant declines in interest in faculty careers at research universities between Ph.D. entry and completion (mean = 2.93 at completion v. 3.47 at entry; p<0.0001); significant, yet smaller, declines in interest in faculty careers at teaching-intensive universities (mean = 2.79 at completion v. 2.97 at entry; p<0.0001); small increases in interest in research careers outside of academia (mean = 3.24 at completion v. 3.12 at entry; p = 0.02), and a significant increase in interest in non-research careers (mean = 3.00 at completion v. 2.14 at entry; p<0.0001) (Fig. 1A).

### Career Interest Trends Intensified for Women from URM Backgrounds

To assess the extent to which average interest patterns for the overall sample were shared or distinct across social identity, data were disaggregated and interest profiles were analyzed by race/ethnicity, gender, and their intersection. Males from well-represented (WR) backgrounds (i.e. White and Asian) are referred to as WRM (25% of sample); males from URM backgrounds (i.e. Black/African-American, Hispanic/Latino, American Indian/Alaska Native, or Native Hawaiian/Pacific Islander) are referred to as URMM (5.8% of sample); females from WR backgrounds are referred to as WRF (53% of sample); and females from URM backgrounds are referred to as URMF (12.6% of sample) (Fig. 1B). Of note, our sample included 276 scientists from URM backgrounds (17.7%)—the largest sample of URM scientists in the last decade to our knowledge [32]—allowing analysis of whether or how their career interests and training experiences differ from their WR colleagues.

When comparing interest in faculty careers at research-intensive universities, there were notable differences in average interest across social identity groups at each time point (Fig. 1Ci). All groups reported declines in interest over time (p<0.001, paired t-tests). However, at entry, men from all backgrounds reported greater interest in faculty careers at research universities than women from all backgrounds (with differences ranging from 0.34–0.74 units; p<0.001). When comparing women at entry, URMF reported lower interest than WRF (0.31 units; p = 0.04) and had the lowest interest of any social-identity group. Further, women continued to report lower interest in faculty careers at research institutions than WRM over time, with URMF reporting lower interest than all other social groups at each time point. In contrast, there were no statistically significant group differences at any time point with respect to interest in faculty careers at teaching-intensive universities (Fig. 1Cii), or research careers outside of academia (Fig. 1Ciii). Thus, the pattern of lower interest from women generally, and URM women specifically, seen in faculty careers at research universities was not seen in all faculty careers or in all research-based careers; it was unique to interest in faculty careers at research universities. Group differences again emerged in interest in non-research careers (Fig. 1Civ). There were no group differences in interest at the start of graduate training, and all groups reported significantly increased interest at Ph.D. completion (p<0.0001, paired t-tests). However, on average, URMF reported higher interest at Ph.D. completion in non-research careers than any other group (0.35–0.49 units greater; p<0.04).

In addition to comparing differences in average interest across groups, we assessed each scientist's change in interest between the beginning and end of their graduate training. Aggregating these data across social-identity groups showed no statistically significant differences in the average magnitude of change in interest in faculty careers at teaching-intensive universities or for research careers outside of academic environments; however, there were differences in the magnitude of interest change in faculty careers at research universities and non-research careers (Fig. 1D). WRM had the smallest decline in interest in faculty careers at research institutions (−0.42 units), followed by WRF (−0.62 units), URMM (−0.75 units), and URMF (−0.75 units; p = 0.046 compared to WRM; Fig. 1Di). On average, URMF had the largest increase in interest in non-research careers (1.33 units), with all other groups having smaller, statistically indistinguishable increases (0.63–0.84 units; p<0.001 for all groups compared to URMF; Fig. 1Div). Thus, on average, URMF showed an intensification of the trends of decreased interest in faculty careers at research-intensive universities (relative to WRM), and increased interest in non-research careers (relative to all other groups).

### Predictors of High Career Pathway Interest at Ph.D. Completion

While these descriptive analyses show group differences in career pathway interest at Ph.D. completion, they do not account for training experiences, measures of research productivity [33], or access to mentoring [34], which may also have an influence on career interests. Multiple logistic regression was used to model the likelihood that respondents would express high interest in each career path (i.e. answering 4 or 5 on the 5-point interest scale) at Ph.D. completion. Each model included three classes of explanatory variables: (i) personal dispositions (level of interest in the career path at Ph.D. entry, intention to pursue a faculty career at Ph.D. entry, confidence in ability as an independent researcher); (ii) objective performance measures (rate of first-author publications, h-index, time-to-Ph.D., institution type); and (iii) graduate training experiences (sense of belonging, faculty advisor interactions, departmental career development). These analyses are shown in table 1.

**Table 1:** Multiple Logistic Regression of Factors Associated with Reporting High Interest in Each Career Pathway at Ph.D. Completion.

		Career Pathway			
Covariate Class	Covariate	Faculty, Research Intensive	Faculty, Teaching Intensive	Research Career, non-academic	Non-Research Career
Social Identity	Well represented, Male (WRM)	Reference	Reference	Reference	Reference
	Underrepresented Minority, Male (URMM)	0.60 (0.36–0.98)*	0.89 (0.49–1.64)	1.06 (0.68–1.67)	1.03 (0.54–1.94)
	Well represented, Female (WRF)	0.64 (0.47–0.89)*	1.10 (0.80–1.50)	1.00 (0.74–1.32)	1.02 (0.75–1.38)
	Underrepresented Minority, Female (URMF)	0.46 (0.30–0.71)**	1.05 (0.65–1.70)	0.75 (0.46–1.22)	1.93 (1.28–2.90)*
Personal Disposition	High Interest in Career Pathway at Ph.D. Entry (High v. Low)	5.16 (3.68–7.23)**	7.29 (5.60–9.51)**	4.89 (3.74–6.39)**	10.76 (7.41–15.62)**
	Intention to Pursue Faculty Career at Ph.D. Entry	1.21 (1.06–1.37)*	1.29 (1.13–1.46)*	0.88 (0.79–0.97)*	0.90 (0.80–1.02)
	Confidence in Ability as Independent Researcher	1.64 (1.41–1.92)**	0.82 (0.71–0.96)**	1.17 (1.02–1.34)*	0.76 (0.66–0.87)**
Objective/Performance	First-Author Publication Rate (Publications/Year in Research)	2.33 (1.52–3.58)**	1.00 (0.62–1.64)	0.86 (0.59–1.26)	0.64 (0.43–0.94)*
	h-index	1.00 (0.98–1.04)	1.00 (0.97–1.04)	1.01 (0.98–1.05)	0.96 (0.92–1.01)
	Time-to-Ph.D. (Years)	0.93 (0.81–1.08)	1.03 (0.90–1.18)	0.99 (0.87–1.13)	1.03 (0.92–1.15)
	Ph.D. at Top 50 Research University (Yes/No)	0.63 (0.45–0.86)*	0.94 (0.71–1.26)	0.82 (0.64–1.03)	1.12 (0.86–1.47)
Graduate Training Experiences (Belonging, Advisor Support, Career Development)	Intellectual Belonging, Research Group	1.20 (0.95–1.52)	0.95 (0.76–1.20)	1.02 (0.81–1.28)	1.19 (0.97–1.46)
	Social Belonging, Research Group	1.00 (0.82–1.21)	0.91 (0.74–1.11)	0.94 (0.79–1.12)	1.02 (0.87–1.20)
	Intellectual Belonging, Department	0.89 (0.75–1.05)	1.03 (0.85–1.25)	0.93 (0.80–1.09)	0.96 (0.78–1.17)
	Social Belonging, Department	1.07 (0.90–1.27)	1.11 (0.94–1.31)	1.12 (0.97–1.30)	1.05 (0.88–1.26)
	Advisor Invested In Career Advancement	1.33 (1.16–1.52)**	1.15 (0.99–1.33)	0.90 (0.78–1.03)	0.90 (0.78–1.02)
	Advisor equally supportive of students pursuing academic & non-academic career paths	0.96 (0.85–1.09)	0.81 (0.72–0.93)*	1.09 (0.98–1.21)	0.93 (0.83–1.03)
	Department offered structured career development	1.01 (0.88–1.16)	1.11 (0.98–1.27)	0.94 (0.81–1.08)	0.92 (0.81–1.04)
	Department equally supportive of students pursuing academic & non-academic career paths	1.23 (1.04–1.46)*	1.12 (0.96–1.32)	1.13 (0.98–1.30)	0.97 (0.83–1.14)

Adjusted Odds Ratios (and 95% Confidence Interval) Shown.

* p<0.05.

** p<0.0001.

For all career pathways, a high level of starting interest predicted high interest at Ph.D. completion (adjusted odds ratios (OR) ranged from 4.89–10.76, p<0.001). Stronger intentions to pursue a faculty career at Ph.D. entry were positively associated with high interest in pursuing faculty careers at research-intensive (OR: 1.21, 95% CI: 1.06–1.37, p = 0.003) and teaching intensive universities (OR: 1.29, 95% CI: 1.13-1.46, p<0.001), and were negatively associated with high interest in pursuing a research career outside of academia (OR: 0.88, 95% CI: 0.79–0.97, p = 0.012). Higher research self-efficacy was positively associated with interest in faculty careers at research-universities (OR: 1.64, 95% CI: 1.41–1.92, p<0.001), and research careers outside of academia (OR: 1.17, 95% CI: 1.02–1.34, p = 0.023), and was negatively associated with high interest in faculty careers at teaching-intensive universities (OR: 0.82, 95% CI: 0.71–0.96, p = 0.012) and in non-research based careers (OR: 0.76, 95% CI: 0.66–0.87, p<0.001).

With regard to objective measures, higher as first-author publication rate was positively associated with interest in faculty careers at research-intensive universities (OR: 2.33, 95% CI: 1.52–3.58, p<0.001), negatively associated with high interest in non-research based careers (OR: 0.64, 95% CI: 0.43–0.94, p = 0.024), and not associated with interest in faculty careers at teaching-intensive institutions or in research careers outside of academia. Attending a top 50-research university was negatively associated with interest in a faculty career at a research-intensive university (OR: 0.63, 95% CI 0.45–0.86, p = 0.005), and in this sample neither h-index, nor time to degree predicted interest in any of the career pathways.

With respect to graduate training experiences, higher levels of reported advisor career investment were positively associated with high interest in faculty careers at research-intensive universities (OR: 1.33, 95% CI: 1.16–1.52, p<0.001), as were higher levels of departmental support for students pursuing either academic or non-academic careers (OR: 1.23, 95% CI: 1.04–1.46, p = 0.012). Perceived sense of “belonging”—either intellectually or socially to a scientist's research group or department—was not associated with career pathway interests. Collectively, these data show that personal attributes—such as career interest at Ph.D. entry, and research self-efficacy—were associated with high levels of interest at Ph.D. completion in each career pathway. Additionally, publication record and advisor investment positively predicted high interest in pursuing a faculty career at a research-intensive university, while receiving a Ph.D. from a Top 50 university negatively predicted interest in this career pathway. However, as these are cross-sectional data, neither causality nor directionality can be determined from these associations [35], [36].

### Disparate Career Interest Profiles at Ph.D. Completion by Social Identity

**Figure 2:**
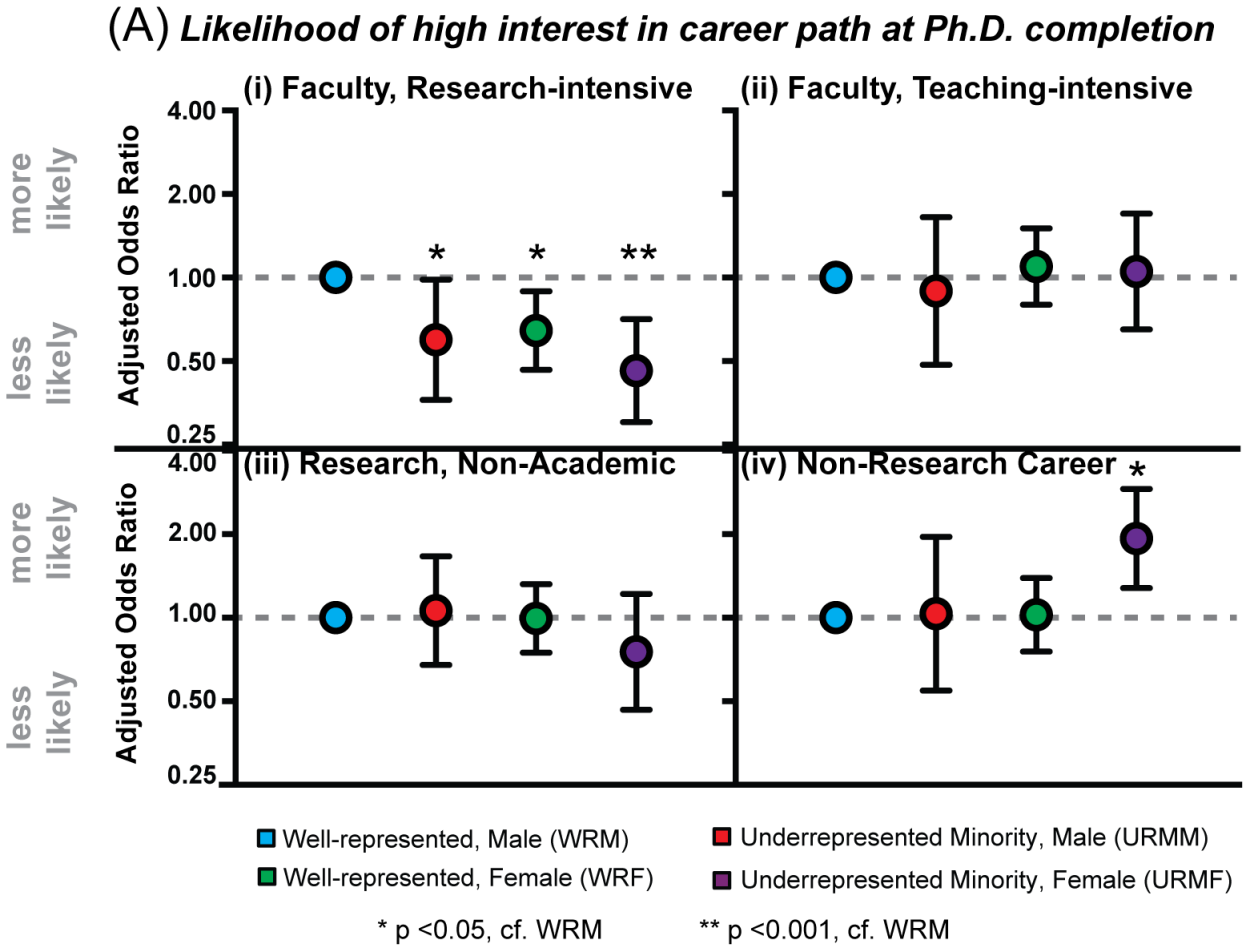
Disparate Career Interest Profiles Across Social Identity. (A) Plot of adjusted odds-ratio (circle) and 95% confidence interval, with males from well-represented racial/ethnic backgrounds (WRM) as the reference group, showing likelihood of expressing high interest (i.e. 4 or 5 on the 5-point interest scale) in four career paths at Ph.D. completion: (i) faculty at a research-intensive university, (ii) faculty at teaching-intensive university, (iii) a non-academic research career, and (iv) a non-research career. Odds ratios are adjusted for personal dispositions (level of interest in career path at Ph.D. entry, intention to pursue faculty career at Ph.D. entry, and confidence in ability as an independent researcher), objective measures (rate of first-author publications, h-index, time-to-Ph.D., Ph.D. institution type), and graduate training experiences (socialization measures, advisor interactions, and career development experiences).

Logistic regression analysis also showed that after controlling for personal dispositions, objective measures, and graduate training experiences, there were significant differences by social identity in the likelihood of expressing high interest in faculty careers at research-intensive universities (Fig. 2A). All groups were statistically less likely than WRM to report high interest in a faculty career at a research-intensive university (URMM OR: 0.60, 95% CI: 0.36–0.98, p = 0.043; WRF OR: 0.64, 95% CI: 0.47–0.89, p = 0.008; URMF OR: 0.46, 95% CI: 0.30–0.71, p<0.001; Fig. 2Ai). That is, after controlling for background characteristics, objective performance, graduate training experiences, and self-efficacy, on average, URMM were 40% less likely, WRF were 36% less likely, and URMF were 54% less likely to express high interest in faculty careers at research universities after completing their Ph.D. as compared to WRM. There were no statistically significant differences by social identity in the likelihood of high interest in faculty careers at a teaching-intensive university, or research careers outside of academia (Fig. 2Aii, iii). With respect to high interest in careers outside of research, URMM (OR 1.03, 95% CI: 0.55–1.95, p = 0.91) and WRF (OR: 1.02, 95% CI: 0.75–1.39, p = 0.88) were comparable to WRM, while URMF were almost twice as likely to report high interest (OR: 1.93, 95% CI: 1.28–2.90, p = 0.002 relative to WRM). Thus, after controlling for multiple factors believed to have an influence on career development, there were disparate career interest profiles by social identity at Ph.D. completion, with all groups less likely than WRM to report high interest in faculty careers at research-intensive universities (URMF being least likely), and URMF more likely to report high interest in non-research careers. These trends remain when accounting for the current work position of the respondents (S4 Table).

## Discussion

Policy makers have focused attention in recent years on two interrelated aspects of the biomedical workforce. Broadly, there has been focus on adjusting graduate and postdoctoral training to better prepare trainees for a career landscape in which an estimated 11–26% Ph.D. biomedical scientists progress to tenure-track professorships [17], [24], [37]. Alongside these efforts, there remains a focus on enhancing research workforce diversity generally, and faculty diversity specifically, because of the benefits diversity brings with respect to problem solving, and the positive effects of professorial diversity on the persistence of women and URMs in science [5]–[7]. While interventions to broaden participation in the workforce are often based on the assumption that if women and URMs progress through the system, demonstrate research productivity [33], and are well mentored [34], they will naturally choose faculty careers, data on whether and how career preferences differ across social identity for biomedical science Ph.Ds. remains lacking. This study presents survey data on the career development and graduate training experiences of a diverse group of recent, American biomedical science graduates—and to our knowledge, the largest sample of scientists from URM backgrounds in the past decade [32]—and can serve to inform ongoing and future efforts.

These findings add to the growing literature regarding the changing career preferences of early career scientists [18], [19]. In agreement with earlier studies, we found that on average, scientists from all backgrounds reported less interest in faculty careers (particularly for those at research-intensive universities), and increased interest in careers outside of research over time. Additionally, we examined whether and how career interest trends differ based on race/ethnicity, gender, and their intersection. Our work shows that even after controlling for multiple factors believed to have an influence on career development (e.g., self-efficacy, objective and performance measures, and advisor interactions), there are disparate career interest profiles at Ph.D. completion for certain career paths. Specifically, women (WR and URM) and URM men were less likely to report high interest in faculty careers at research-intensive universities relative to WRM, with URMF showing lower interest than all groups. Moreover, despite equal interest among social groups in non-research careers at Ph.D. entry, URMF were much more likely than other groups to express high interest in these careers at Ph.D. completion.

The unique patterns observed among URM women suggest that the application of an intersectional lens—i.e. consideration of how race/ethnicity and gender act simultaneously to shape experiences—would be fruitful in efforts that aim to increase faculty and workforce diversity [38]–[40]. Women account for 58% the Ph.Ds. awarded to biological scientists from URM backgrounds [4]. Thus, to make progress and promote inclusion, initiatives focused on increasing faculty and workforce diversity must consider how the experiences and career development patterns of women of color are unique and differ from well-represented women and men from underrepresented backgrounds.

**Figure 3:**
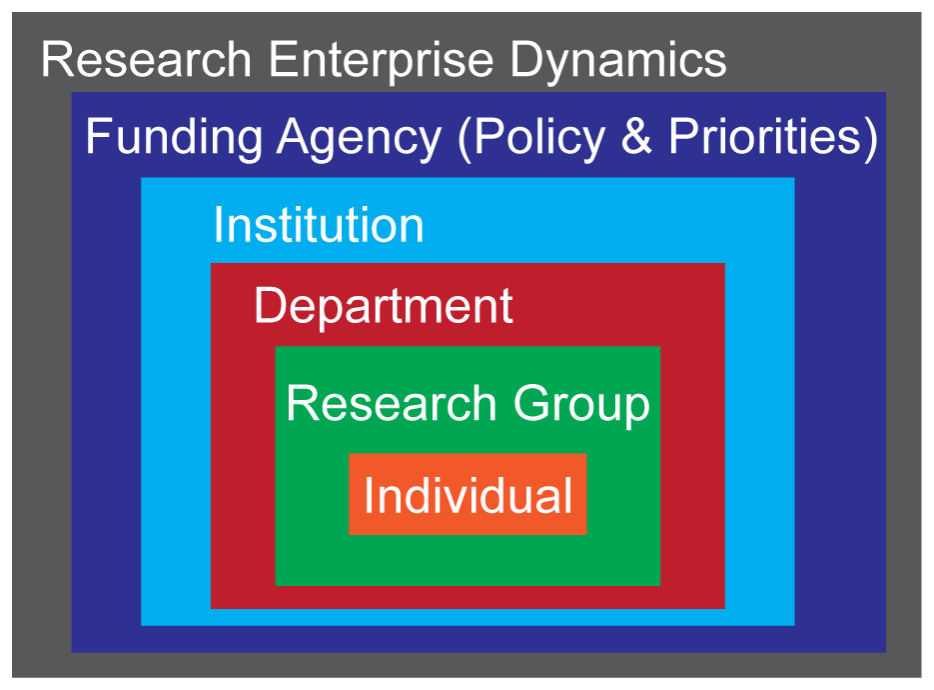
Ecological Conceptualization of Factors Impacting Career Development of Biomedical Scientists. As disparities in career interest at Ph.D. completion by social identity persist when controlling for productivity, self-efficacy, advisor interactions, and other determinants of career choice, we propose that workforce development and diversity efforts utilize an ecological framework that takes into account the multiple agents that can influence the career decision-making process. These include individual level variation, research group and advisor, department and institutional training environment, pressures exerted by funding agencies, and the broader dynamics in the research enterprise.

These data capture the phenomenon of disparate career interest profiles, but are not able to fully explain why these trends exist. To better understand the mechanisms underlying career choice generally, and these disparate outcomes specifically, we are interviewing a subset of respondents from all social backgrounds who report diverse career interests and trajectories. We are utilizing an ecological framework [41] that aims to take into account multiple factors that can potentially act to influence individual decision-making (e.g. personal dispositions, research group and advisor, department and institution culture, funding agency policy and priorities, and broader systemic dynamics), including those over which individual scientists have no direct control, and can only be modified through policy (Fig. 3).

The data presented in this study are not meant to suggest that all Ph.D. recipients should express interest in being faculty members. There are many career paths for Ph.D. biomedical scientists [42]. At the same time, diversity in the nation's science faculties and research workforce has remained a priority at the institutional and federal levels [11]–[13], [43] because of the benefits with respect to creativity in problem solving, student retention, and student learning [6]–[10]. Part of the underrepresentation of certain populations in some disciplines can be attributed to the pool of available talent. However, these data strongly suggest that policy solutions that focus principally on increasing the supply of talent from underrepresented backgrounds (often referred to as increasing the “pipeline”), will not be adequate for significantly enhancing representation on science faculties, as evidenced by the disparate career interest patterns across social identity in recent Ph.D. graduates. In addition to a more nuanced view of career interest formation, these data suggest that efforts to diversify the professoriate should also consider the influences of the broader dynamics and reward structures operating at the institutional and systemic levels, and whether/how they might exert differential selective pressures across social identity [1], [15], [21], [44]–[47]. Ultimately, more qualitative work addressing not only how, but *why* individual interests change, and whether there are unique factors impacting underrepresented groups is needed so that policy makers can more effectively design interventions and strategies to strengthen the biomedical enterprise through enhanced workforce and professorial diversity.

## Supporting Information

S1 Figure **Survey Instrument.**
(PDF)

S1 Table **Ph.D. granting institutions of biomedical science Ph.D. survey respondents.**
(XLSX)

S2 Table **Current positions of biomedical science Ph.D. survey respondents.**
(XLSX)

S3 Table **Ph.D. disciplines of biomedical science survey respondents.**
(XLSX)

S4 Table **Multiple logistic regression showing factors associated with biomedical graduates reporting high interest in each career pathway at Ph.D. completion (taking into account current position).** Adjusted Odds Ratios (and 95% Confidence Interval) Shown.
(XLSX)
