# Supplementary material for: Biomedical Science Ph.D. Career Interest Patterns by Race/Ethnicity and Gender
Source: PLoS One. 2014 Dec 10;9(12):e114736. doi: 10.1371/journal.pone.0114736 (PMC4262437; doi:10.1371/journal.pone.0114736)
Supplement: S1 Figure — Survey Instrument. (PDF) [file pone.0114736.s001.pdf]

**Project Title**	**Assessing the Determinants of Career Choice in Recent Ph.D. Completers in the Biomedical, Behavioral and Physical Sciences**
**Purpose of the Study**	***This research is being conducted by Kimberly Griffin, University of Maryland, College Park and Kenneth Gibbs, AAAS Science & Technology Policy Fellow. We are inviting you to participate in this research project because you have completed doctoral study in the biomedical, behavioral or physical sciences. The purpose of this research project is to gain a deeper understanding of the factors, forces, and structures related to the career choices of recent PhD scientists in these fields.***
**Procedures**	***The procedures involve participation in an on-line questionnaire, which will take approximately 5-10 minutes to complete. Questions include "When I started my PhD, I had a clear career goal" and "As a graduate student, I belonged to the intellectual community of my research group." All participants will be entered into a drawing where you can win one of ten, $100 Amazon.com gift cards.***
**Potential Risks and Discomforts**	***There may be some risks from participating in this research study, including discomfort from the questions asked or experiencing sadness or negative emotions associated with certain experiences.***
**Potential Benefits**	***Participants will receive no direct benefits from participation. We hope that, in the future, other people might benefit from this study through improved understanding of how individuals experience their training and make decisions about their career paths.***
**Confidentiality**	***Any potential loss of confidentiality will be minimized by storing data on a password protected computer in a locked office. All participants will be assigned pseudonyms, which will be linked on one password-protected document.***<br><br>***If we write a report or article about this research project, your identity will be protected to the maximum extent possible. Your information may be shared with representatives of the University of Maryland, College Park or governmental authorities if you or someone else is in danger or if we are required to do so by law.***
**Medical Treatment**	***The University of Maryland does not provide any medical, hospitalization or other insurance for participants in this research study, nor will the University of Maryland provide any medical treatment or compensation for any injury sustained as a result of participation in this research study, except as required by law.***
**Right to Withdraw and Questions**	***Your participation in this research is completely voluntary. You may choose not to take part at all. If you decide to participate in this research, you may stop participating at any time. If you decide not to participate in this study or if you stop participating at any time, you will not be penalized or lose any benefits to which you otherwise qualify.***<br><br>***If you decide to stop taking part in the study, if you have questions, concerns, or complaints, or if you need to report an injury related to the research, please contact the investigator:***<br>***Kimberly A. Griffin***<br>***3214 Benjamin Building, College of Education, University of Maryland, College Park, MD 20740;***<br>***kgriff29@umd.edu,* (301) 405-2858**
**Participant Rights**	***If you have questions about your rights as a research participant or wish to report a research-related injury, please contact:***<br><br>**University of Maryland College Park**<br>**Institutional Review Board Office**<br>**1204 Marie Mount Hall**

	**College Park, Maryland, 20742**<br>**E-mail: irb@umd.edu**<br>**Telephone: 301-405-0678**<br><br>***This research has been reviewed according to the University of Maryland, College Park IRB procedures for research involving human subjects.***
**Statement of Consent**	***By proceeding, you indicate that you are at least 18 years of age; you have read this consent form or have had it read to you; your questions have been answered to your satisfaction and you voluntarily agree to participate in this research study. You will receive a copy of this signed consent form. If you agree to participate, please continue to the next screen and begin completion of the questionnaire.***<br><br>***If you agree to participate, please continue to the next screen and begin completion of the questionnaire.***

>>

Are you a US Citizen or Permanent Resident of the United States?

- Yes
- No

Did you complete a PhD in the biomedical, behavioral, or physical sciences between January 1, 2007 and December 31, 2012?

- Yes
- No

<< >>

These questions focus on your career intentions when you began your graduate studies.

Please consider how strongly you agree or disagree with the following statements:

	Strongly Disagree	Disagree	Neither Agree nor Disagree	Agree	Strongly Agree
When I began my PhD, I had a clear career goal	○	○	○	○	○
When I began my PhD, I intended to pursue a faculty career	○	○	○	○	○
When I began my PhD, I was knowledgeable about the various career options available to a person with a PhD in my discipline	○	○	○	○	○

Please rate your interest in pursuing each of the following career pathways at the beginning of your PhD training

	Not Knowledgeable	No Interest	Low Interest	Moderate Interest	Interest	Strong Interest
Faculty at a research-intensive university	○	○	○	○	○	○
Faculty at a teaching-intensive university	○	○	○	○	○	○
Research career, non-academic (industry, pharmaceutical, biotech, government, start-up, etc.)	○	○	○	○	○	○
Non-research career (consulting, policy, science writing, patent law, business, etc.)	○	○	○	○	○	○

<< >>

These questions focus on your experiences during graduate school. How strongly do you agree or disagree with the following statements about your graduate training?

	Strongly Disagree	Disagree	Neither Agree nor Disagree	Agree	Strongly Agree
I belonged to the intellectual community of my research group	○	○	○	○	○
I belonged to the social community of my research group	○	○	○	○	○
I belonged to the intellectual community of my department	○	○	○	○	○
I belonged to the social community of my department	○	○	○	○	○
My graduate advisor was invested in my career advancement	○	○	○	○	○
My graduate advisor was equally invested in the career advancement of all graduate students in the research group	○	○	○	○	○
My graduate advisor was equally supportive of students pursuing academic and non-academic career paths	○	○	○	○	○
My department offered structured opportunities to explore a variety of career pathways (academic and non-academic)	○	○	○	○	○
My department was equally supportive of students pursuing academic and non-academic career paths	○	○	○	○	○

Please rate your level of agreement with the following statements when you completed your PhD

	Strongly Disagree	Disagree	Neither Disagree nor Agree	Agree	Strongly Agree
When I completed my PhD, I had a clear career goal	○	○	○	○	○
When I completed my PhD, I intended to pursue a faculty career	○	○	○	○	○
When I completed my PhD, I was knowledgeable about the various career options available to a person with a PhD in my discipline.	○	○	○	○	○

Please rate your interest in pursuing each of the following career pathways at the completion of your PhD training

	Not Knowledgeable	No Interest	Low Interest	Moderate Interest	Interest	Strong Interest
Faculty at a research-intensive university	○	○	○	○	○	○
Faculty at a teaching-intensive university	○	○	○	○	○	○
Research career, non-academic (industry, pharmaceutical, biotech, government, start-up, etc.)	○	○	○	○	○	○
Non-research career (consulting, policy, science writing, patent law, business, etc.)	○	○	○	○	○	○

Are you currently a postdoctoral researcher (postdoc)?

- Yes
- No

Have you ever been a postdoctoral researcher (postdoc)?

- Yes
- No

<< >>

Including all postdoctoral positions you've held (current and previous), how long have you been in/were you in postdoctoral training?
Please enter your response in years, using numerical values only - e.g, 1 year and 6 months should be entered as 1.5

Including your current postdoctoral position, how many postdoctoral positions have you held?
Please only use numerical values in your response

Please answer the questions below about the nature of your postdoctoral training.

- **If you <u>have had multiple postdoctoral positions</u>, please answer the following questions based on your current or most recent postdoctoral position.**
- **If you are <u>not currently in a postdoctoral position but were previously</u>, please answer the following questions based on your last postdoctoral experience.**

How strongly do you agree or disagree with the following statements about your postdoctoral training?

	Strongly Disagree	Disagree	Neither Agree nor Disagree	Agree	Strongly Agree
As a postdoc, I belong/belonged to the intellectual community of my research group	○	○	○	○	○
As a postdoc, I belong/belonged to the social community of my research group	○	○	○	○	○
My postdoctoral advisor is/was invested in my career advancement	○	○	○	○	○
My postdoctoral advisor is/was equally invested in the career advancement of all postdocs in the research group	○	○	○	○	○
My postdoctoral advisor is/was equally supportive of postdocs pursuing academic and non-academic career paths	○	○	○	○	○
My postdoctoral institution offers/offered structured opportunities to explore a variety of career pathways	○	○	○	○	○
My institution offers/offered structured opportunities to develop non-research skills	○	○	○	○	○

<< >>

The following questions focus on your current career aspirations and how your career interests have changed over the course of your training.

Please rate your agreement with the following statements about your current career aspirations and skills:

	Strongly DIsagree	Disagree	Neither Agree nor Disagree	Agree	Strongly Agree
I am confident in my abilities as an independent researcher	○	○	○	○	○
I have a clear career goal	○	○	○	○	○
I intend to pursue a faculty career	○	○	○	○	○
I am knowledgeable about the various career options available to a person with a PhD in my discipline	○	○	○	○	○

Please rate your current interest in the following career pathways

	Not Knowledgeable	No Interest	Low Interest	Moderate Interest	Interest	Strong Interest
Faculty at a research-intensive university	○	○	○	○	○	○
Faculty at a teaching-intensive university	○	○	○	○	○	○
Research career, non-academic (industry, pharmaceutical, biotech, government, start-up, etc.)	○	○	○	○	○	○
Non-research career (consulting, policy, science writing, patent law, business, etc.)	○	○	○	○	○	○

Since your entry into graduate school have you become more or less likely to pursue each of the following career paths

	Much Less Likely	Less Likely	Neither More nor Less Likely	More Likely	Much More Likely
Faculty at a research-intensive university	○	○	○	○	○
Faculty at a teaching-intensive university	○	○	○	○	○
Research career, non-academic (industry, pharmaceutical, biotech, government, start-up, etc.)	○	○	○	○	○
Non-research career (consulting, policy, science writing, patent law, business, etc.)	○	○	○	○	○

<< >>

Please indicate whether you have participated in or received support from any of the following programs, funding sources, or conferences <u>over the course of your scientific training (i.e. pre-college through postdoctoral)</u>?

Please mark all that apply

Have you received support or participated in programs funded by the National Institutes of Health (NIH)?

- ☐ Supported as a research assistant on my PI's NIH grant
- ☐ I have been a trainee (e.g. summer student, graduate student, postdoc) in an NIH lab
- ☐ Awarded an NIH Fellowship (e.g., National Research Service Awards or Career Development Awards)
- ☐ Supported on an NIH institutional training grant (e.g. T32)
- ☐ Participated in NIH training or institutional development program (e.g. MARC, RISE, IMSD, USP, PREP, Bridges to the Baccalaureate, Bridges to the Doctorate)
- ☐ Other NIH funding, training, or support
- ☐ I have not received support from or participated in NIH programs

Have you received support or participated in programs funded by the National Science Foundation (NSF)?

- ☐ Supported as a research assistant on my PI's NSF grant
- ☐ Awarded an NSF Fellowship (e.g., Graduate Research Fellowship program, Graduate STEM Fellows in K-12 Education, Postdoctoral Research Fellowships in Biology)
- ☐ Participated in a NSF training or institutional development program (e.g., REU, IGERT, AGEP, LSAMP, CREST, HBCU-UP, TCUP)
- ☐ Other NSF funding, training, or support
- ☐ I have not received support from or participated in NSF programs

Have you received support or participated in programs funded by other sources?

- ☐ ABRCMS: Annual Biomedical Research Conference for Minority Students
- ☐ AISES: American Indian Science and Engineering Society
- ☐ Ford Foundation Fellowship (Predoctoral, Dissertation, or Postdoctoral)
- ☐ The Leadership Alliance Summer Research Early Identification Program (SR-EIP)
- ☐ McNair Scholars
- ☐ Meyerhoff Scholars Program
- ☐ NSBE: National Society of Black Scientists and Engineers
- ☐ SACNAS: Society for the Advancement of Chicanos and Native Americans in Science
- ☐ UNCF-Merck Science Initiative (Undergraduate, Graduate, Postdoctoral)
- ☐ Other funding, training, or support

<< >>

Please answer the following demographic questions. Again, all information will be kept confidential and will be used for research purposes only.

What is your gender?

- ☐ Female
- ☐ Male
- ☐ Transgender
- ☐ Decline to State

Were you born in the United States?

- ○ Yes
- ○ No

How would you classify your racial/ethnic identity (please mark all that apply)

- ☐ American Indian/Alaska Native
- ☐ Asian/Asian American
- ☐ Black/African American
- ☐ Hispanic/Latino
- ☐ Native Hawaiian/Pacific Islander
- ☐ White
- ☐ Other
- ☐ Decline to State

From which institution did you receive your undergraduate degree?

What year did you complete your undergraduate degree?

From what institution did you receive your graduate degree?

What year did you complete your graduate degree?

In what discipline/academic program did you complete your graduate degree?

How many years were you in graduate training (i.e. start of program to conferral of degree)?
Please enter your response in years, using numerical values only - e.g, 1 year and 6 months should be entered as 1.5

Please list all institutions at which you have engaged in postdoctoral training. Please list the most recent institution first, and separate institutions by commas.

What degree did you attain?

- Ph.D.
- M.D./Ph.D.
- Other

What is your title in your current position?

How long have you been in your current position?
Please enter your response in years, using numerical values only - e.g, 1 year and 6 months should be entered as 1.5

Are you willing to be contacted about participating in an interview about your training experiences? All participants will receive a $25 Amazon gift card.

- Yes
- No

<< >>

Thank you for completing this questionnaire! All participants who have completed the questionnaire by 11:59 PM on December 15, 2012 are eligible to receive one of ten, $100 Amazon.com giftcards. Please provide us with your contact information to be in touch about the results.

First Name

Last Name

Email Address

<< >>
